# Urinary stone analysis at the Military Hospital of Marrakech: Fourier transform infrared spectroscopic study

**DOI:** 10.11604/pamj.2025.51.78.48414

**Published:** 2025-07-24

**Authors:** Kawtar Benkhaldoun, Errim Ahl Cheikh, Abderrahman Boukhira

**Affiliations:** 1 Biochemistry Department of the Avicenne Military Hospital, Faculty of Medicine and Pharmacy, Marrakech, Morocco

**Keywords:** Urinary stones, composition, Fourier transform infrared spectroscopy

## Abstract

Urolithiasis is a growing global health concern, influenced by a combination of genetic, environmental, and lifestyle factors. Accurate analysis of urinary stone composition is essential for effective diagnosis, treatment, and recurrence prevention. Fourier transform infrared (FTIR) spectroscopy has become a gold standard technique for stone analysis due to its precision and efficiency. This study aimed to evaluate the biochemical composition of urinary stones using FTIR spectroscopy and to identify trends related to patient demographics at the Avicenne Military Hospital in Marrakech. A total of 136 urinary stones were collected from patients between February 2020 and November 2023. Stone samples were analyzed using FTIR spectroscopy, and their composition was correlated with patient age and sex. Only the predominant constituent of each stone was considered for classification. The patient cohort included 82 men and 54 women, with an average age of 52.9 years. Calcium oxalate was the most prevalent component (35.3%), followed by struvite (20.5%), carbapatite + cystine (19.1%), and urate (13.2%). Mixed stones were common, with the majority containing two or more constituents. Calcium oxalate stones were more frequent in men, while struvite and urate stones were more associated with women and older age groups. Variations in stone composition were observed across different age and sex groups, reflecting underlying metabolic and infectious factors. Fourier transform infrared spectroscopy offers a reliable, rapid, and non-destructive method for analyzing urinary stone composition. Understanding the chemical makeup of stones provides essential insights for individualized patient management and targeted prevention strategies. This study highlights the importance of routine stone analysis in optimizing clinical care and reducing recurrence rates.

## Introduction

Urolithiasis, or the development of urinary calculi, represents a significant health issue with a multifactorial origin involving genetic predisposition, environmental influences, and lifestyle-related factors [1]. Its prevalence has been increasing worldwide over the past few decades [2-4]. Several types of urinary lithiasis have been described according to their physicochemical constituents and/or their morphology [5]. Understanding stone composition is crucial for developing a treatment strategy, defining the underlying cause, and preventing recurrence [2]. Infrared spectroscopy has become widely utilized in clinical practice and appears to be a powerful analytical and effective method for identifying and quantifying various constituents of urinary stones, including calcium, oxalate, uric acid, phosphate, acid, and others [6]. The objective of this work is to report the usefulness of the Fourier Transform Infrared spectroscopy technique in the study of the biochemical composition of stones in the Avicenne Military Hospital of Marrakech.

## Methods

We collected a total of 136 urinary stones from 136 patients with lithiasis at the Biochemical Department of Avicenne Military Hospital between February 2020 and November 2023. The study included 136 patients-82 males and 54 females-aged between 19 and 80 years. Individuals with missing information on age or gender were excluded from the analysis. The stones were obtained from patients through various methods, including spontaneous passage, passage after extracorporeal shockwave lithotripsy, or during surgical removal. To ensure diverse samples, we selected only the initial calculation. Fourier transform infrared spectroscopy (FTIR) was used to analyze the stones that were presented to the stone analysis laboratory, avoiding duplicates from subsequent treatment sessions. Stone analysis was performed using Fourier Transform Infrared (FTIR) spectroscopy. Initially, the stones were washed with distilled water and thoroughly dried. After being crushed into a fine powder with a mortar and pestle, the stone samples were oven-dried at 100°C for 24 hours. A portion of 10 mg was mixed with 300 mg of potassium bromide, pressed manually into a pellet, and then placed in the FTIR spectrophotometer for analysis. Fourier transform infrared spectroscopy (FTIR) was used to analyze the stones that were presented to the stone analysis laboratory, avoiding duplicates from subsequent treatment sessions. Stone analysis was performed using Fourier transform infrared spectroscopy, which was conducted using a JASCO FT/IR-4600 instrument, covering a wave number range from 7800 to 350 cm^-1^. The identification of stone components was carried out by matching the infrared spectra obtained from each sample with a digital database containing reference spectra for both pure and mixed stone types. All analyses were conducted by trained personnel and independently verified to ensure precision and reliability. Considerable investment was made to obtain the JASCO FTIR equipment, and laboratory staff received specialized training to operate it effectively. Today, FTIR remains the preferred method for routine clinical characterization of urinary stone composition, due to its accuracy and efficiency.

## Results

**Population characteristics:** in our study, a total of 136 stones were analyzed from February 2020 to November 2023, with 82 stones from men and 54 from women, resulting in a sex ratio of 1.5. Patients had a mean age of 52.9 years. Age distribution showed that 29 stones originated from individuals aged 19 to 40, 66 from those between 41 and 60 years old, and 41 from patients in the 61 to 80 age group (Table 1).

**Table 1:** characteristics of urinary stones according to the gender of 70 patients

Age (year)	Overall n=136	Male n=82	Female n=54
19-30	5	1	4
31-40	24	16	8
41-50	24	15	9
51-60	42	21	21
61-70	27	20	7
71-80	14	9	5

**Stone composition:** urinary calculi can have a single or multiple constituents. However, for this study, only the predominant compound will be considered. Mixed-type calculi were predominant among the samples analyzed, with 113 stones consisting of two constituents and 19 stones consisting of three or more constituents. Calcium oxalate monohydrate (COM) in association with calcium oxalate dihydrate (COD) represented the most frequent compositional pattern (35 stones), followed by carbapatite/cystine (26 stones), struvite/newberyite (26 stones), ammonium acid urate (AAU)/struvite (11 stones), COM/AAU/carbapatite (7 stones), and AAU/COM (7 stones) (Table 2). Among all stone types, calcium oxalate appeared most frequently, representing 35.3% of cases, followed by struvite (20.5%), carbapatite + cystine (19.1%), urate (13.2%), and other components (11.8%) (Table 3).

**Table 2:** distribution of stones composed of single or multiple components

Stones with 1 component	n	Stones with 2 components	n	Stones with 3 or more components	n
Opaline silica	1	COM/COD	35	COM/AAU/carbapatite	7
		AAU/UAD	2	COM/COD/AAU/carbapatite	2
Aluminium silicate	1	Struvite/Newberyite	26	COM/carbapatite/struvite	7
Penicillin G sediment	1	AAU/COM	7	Calcium/bilirubinate/carbapatite/protein	1
Proteins	1	AAU/Struvite	11	COM/COD/UAM	1
		Carbapatite/cystine	26	COM/COD/AAU	1
		COM/carbapatite	2		
		Newberyite/MSU	4		
COM: calcium oxalate monohydrate; COD: calcium oxalate dihydrate; AAU: ammonium acid urate; UAD: uric acid dihydrate; MSU: monosodium urate, UAM: uric acid monohydrate; Struvite: magnesium ammonium phosphate; carbapatite: Carbonated apatite; newberyite: magnesium hydrogen phosphate trihydrate					

**Table 3:** distribution of the main urinary stone constituents according to the gender of the patients

Composition	Overall n=136	Male n=82	Female n=54
Calcium oxalate (CaOx)	48	30	18
Cystine + carbapatite	26	17	9
Ammonium acid urate (AAU)	18	13	5
Struvite	28	17	11
Carbapatite	8	2	6
Newberyite	4	3	1
Aluminium silicate	1	0	1
Opaline silica	1	0	1
Other	2	0	2

**Stone composition according to sex and age:** in male patients, calcium oxalate stones were the most frequently identified (30 stones), followed by combinations of cystine and carbapatite (17 stones), struvite (17 stones), and ammonium acid urate (AAU) (13 stones), among others. Among females, calcium oxalate also ranked first (18 stones), with struvite (11 stones), cystine/carbapatite (9 stones), and AAU (5 stones) following. While calcium oxalate stones were the most prevalent in both sexes, they were more commonly observed in males. To assess the relationship between age and stone type, patients were categorized into six age groups: 19-30, 31-40, 41-50, 51-60, 61-70, and 71-80 years. The greatest concentration of stone cases occurred between the ages of 51 and 70 for men, and between 41 and 60 for women (Table 1).

## Discussion

Urolithiasis is a global medical issue that has seen an increased prevalence in the past two decades among both men and women. Additionally, there is a high recurrence rate of stone disease within five years, reaching up to 50% [7]. Various studies have revealed that the composition and structure of urinary stones provide valuable information about the stone formation process, influencing treatment plans and prognosis [8]. It is important to emphasize that analyzing the composition and microstructure of urinary stones is crucial as it offers insights into the stone's formation process, informing treatment strategies and prognosis.

Various techniques are currently employed to examine the composition of urinary stones, including both dry and wet chemical assays, X-ray powder diffraction, Raman spectroscopy, and Fourier Transform Infrared (FTIR) spectroscopy [9,10]. Since its introduction in 1955, FTIR has shown high sensitivity and accuracy in detecting stone constituents [11,12]. Thanks to its rapidity and specificity, it has become the most widely adopted technique for routine stone analysis. According to the European Association of Urology Guidelines on Urolithiasis, FTIR spectroscopy and X-ray diffraction are the recommended approaches, whereas traditional chemical tests are now considered obsolete [13]. A key advantage of FTIR lies in its ability to quickly identify a wide range of crystal types. However, effective use of this technique depends heavily on both the operator´s expertise and a thorough understanding of the analytical process.

Urolithiasis primarily affects individuals in their thirties and forties in industrialized nations. Although kidney stone disease has traditionally been more prevalent in men than women, recent research over the past decades indicates that the gap between the sexes is decreasing [14]. This trend may reflect changes in risk factors affecting stone formation differently in men and women. In particular, lifestyle modifications and a rise in obesity rates among women are thought to contribute to this evolving gender distribution [15]. Calcium oxalate (CaOx) was the most common urinary stone component, accounting for 35.3% of stones in both men and women in our study. It was followed by carbapatite + cysteine, struvite, urate, carbapatite, newberyite, and other components. These findings indicate significant variations in stone composition based on gender, age, anatomical localization, and geographical region.

The composition of urinary stones differs across regions globally, with calcium oxalate stones being the most commonly encountered type. Research has demonstrated that dietary intake of oxalate plays a significant role in influencing urinary oxalate levels [16,17]. Since urinary oxalate concentration is a key contributor to the formation of calcium oxalate stones, dietary modifications aimed at reducing oxalate consumption may help patients prevent stone recurrence [18]. Uric acid presence in stones is more common in men, with a rate of 9.6% compared to 3.7% in women. We found that the incidence of urate stones increases with age, which aligns with findings from two large stone analysis studies [19,20]. Furthermore, diabetes and insulin resistance, which are more prevalent with aging, lead to lower urinary pH and an increased likelihood of urate stone formation [21]. Being overweight may also contribute to the occurrence of uric lithiasis [22]. Struvite and carbapatite+cystine are the other prominent components, present in approximately 20.6% and 19.1% of cases, respectively. The remaining components are found in lower percentages. The chemical composition of certain calculi, such as carbapatite, struvite, and others, suggests the presence of urinary tract infection, likely due to hygiene issues, inadequate detection, or improper management [23].

## Conclusion

Differentiating between various crystal forms can offer valuable insights into the activity and mechanisms underlying the formation of stones. A comprehensive understanding of these mechanisms is essential in order to provide suitable individualized treatment and effectively prevent recurrence for each patient. FT-IR spectroscopy has emerged as the optimal method for analyzing stones and is being recognized as the benchmark for stone analysis. It provides clear and precise identification of both major and minor components within the stone. Such detailed information is essential for tailoring appropriate treatment strategies.

### What is known about this topic

- Urolithiasis is a common and recurrent condition, with calcium oxalate being the most frequent component;
- Stone composition varies by region, age, sex, and underlying metabolic and infectious causes;
- Fourier transform infrared spectroscopy is recognized as a reliable and rapid method for identifying urinary stone composition.

### What this study adds

- Provides updated data on urinary stone composition in a Moroccan hospital over 3 years;
- Highlights the prevalence of mixed stone types and their distribution across age and sex groups;
- Confirms the practicality and diagnostic value of the FTIR spectroscopy in routine clinical settings in North Africa.
